# A Novel 3D Convolutional Neural Network-Based Deep Learning Model for Spatiotemporal Feature Mapping for Video Analysis: Feasibility Study for Gastrointestinal Endoscopic Video Classification

**DOI:** 10.3390/jimaging11070243

**Published:** 2025-07-18

**Authors:** Mrinal Kanti Dhar, Mou Deb, Poonguzhali Elangovan, Keerthy Gopalakrishnan, Divyanshi Sood, Avneet Kaur, Charmy Parikh, Swetha Rapolu, Gianeshwaree Alias Rachna Panjwani, Rabiah Aslam Ansari, Naghmeh Asadimanesh, Shiva Sankari Karuppiah, Scott A. Helgeson, Venkata S. Akshintala, Shivaram P. Arunachalam

**Affiliations:** 1 Department of Radiology, Mayo Clinic, Rochester, MN 55905, USA; 2 Department of Biomedical Informatics and Computational Biology, University of Minnesota, Minneapolis, MN 55455, USA; 3 Digital Engineering & Artificial Intelligence Laboratory (DEAL), Mayo Clinic, Jacksonville, FL 32224, USA; 4 Department of Critical Care Medicine, Division of Pulmonary Medicine, Mayo Clinic, Jacksonville, FL 32224, USA; 5 Division of Gastroenterology & Hepatology, Department of Medicine, Johns Hopkins School of Medicine, Baltimore, MD 21218, USA

**Keywords:** gastrointestinal endoscopic video classification, P-scSE3D, 3D convolutional neural network, hyperKvasir dataset, spatiotemporal features, deep learning

## Abstract

Accurate analysis of medical videos remains a major challenge in deep learning (DL) due to the need for effective spatiotemporal feature mapping that captures both spatial detail and temporal dynamics. Despite advances in DL, most existing models in medical AI focus on static images, overlooking critical temporal cues present in video data. To bridge this gap, a novel DL-based framework is proposed for spatiotemporal feature extraction from medical video sequences. As a feasibility use case, this study focuses on gastrointestinal (GI) endoscopic video classification. A 3D convolutional neural network (CNN) is developed to classify upper and lower GI endoscopic videos using the hyperKvasir dataset, which contains 314 lower and 60 upper GI videos. To address data imbalance, 60 matched pairs of videos are randomly selected across 20 experimental runs. Videos are resized to 224 × 224, and the 3D CNN captures spatiotemporal information. A 3D version of the parallel spatial and channel squeeze-and-excitation (P-scSE) is implemented, and a new block called the residual with parallel attention (RPA) block is proposed by combining P-scSE3D with a residual block. To reduce computational complexity, a (2 + 1)D convolution is used in place of full 3D convolution. The model achieves an average accuracy of 0.933, precision of 0.932, recall of 0.944, F1-score of 0.935, and AUC of 0.933. It is also observed that the integration of P-scSE3D increased the F1-score by 7%. This preliminary work opens avenues for exploring various GI endoscopic video-based prospective studies.

## 1. Introduction

Early detection of diseases from video-based medical imaging is an evolving yet underexplored frontier in artificial intelligence (AI). While deep learning (DL) techniques have significantly improved the analysis of static medical images, their applications to video-based imaging for early-stage disease detection remain limited, particularly in leveraging the inherent spatiotemporal features critical for clinical decision-making. Conventional AI studies often rely on still images, overlooking the dynamic temporal dimension present in videos that can provide valuable contextual and motion-related cues for better diagnosis accuracy and early disease recognition [1,2,3]. This gap is especially relevant in procedures like endoscopy, laparoscopy, and ultrasound, where disease progression, lesion evolution, or procedural navigation unfolds over time [4,5,6].

Novel spatiotemporal feature mapping approaches, such as 3D convolutional neural networks (CNNs), offer the potential to bridge this gap by capturing both spatial and temporal dependencies within medical videos [7,8]. Such models can enhance the understanding of disease patterns that might be missed by static frame analysis alone, leading to improved early detection capabilities. However, despite these promising advances, the feasibility and implementation of spatiotemporal DL models in clinical video workflows remain scarce and under-investigated, posing both technical and translational challenges [9].

Gastrointestinal (GI) endoscopic videos provide an ideal use case to explore and demonstrate these possibilities. GI diseases consistently pose challenges to clinical practice, with a significant number of new cases and fatalities occurring annually. Studies [1] show that gastrointestinal, liver, and pancreatic diseases account for more than 54 million annual ambulatory visits and about 3 million hospital admissions in the United States (US). Endoscopy remains a key diagnostic tool in this domain [4], yet its effectiveness relies heavily on the endoscopist’s expertise, resulting in variable diagnostic outcomes [3,5]. A major limitation of most AI studies in GI endoscopy is their reliance on still images. While these studies have shown promising results [8,9,10], they fail to capture the dynamic and complex nature of GI endoscopic procedures, where continuous video streams provide crucial contextual information [11,12,13].

In this feasibility study, we aim to address these gaps by developing a novel 3D CNN-based deep learning model designed for spatiotemporal feature mapping from GI endoscopic videos. Our model specifically classifies upper and lower GI endoscopic videos, leveraging the rich temporal and spatial data present in video streams to improve diagnostic performance. We also introduce a 3D version of the parallel spatial and channel squeeze-and-excitation (P-scSE) module and a residual with parallel attention (RPA) block, coupled with computationally efficient (2 + 1)D convolutions [14,15].

Early video classification relied on combining custom-designed hand-crafted features, like SIFT-3D [16], HOG3D [17], and 3D SURF [18], with advanced machine learning techniques. Recent advancements in deep learning (DL) models have led to DL-based pipelines for video classification. One example is two-stream 2D CNN architecture, which extracts spatial and temporal information from videos through separate networks and then fuses the results, enabling traditional 2D CNNs to handle video data effectively. However, they still struggle with modeling long-term dependencies [19]. To overcome this, recurrent neural networks (RNNs) and its variations are integrated into the CNN architecture. RNN-based methods generally obtain visual percepts by applying a 2D CNN as a feature extractor to the video frames and then inputting the CNN activations into an RNN to capture the temporal variations in the video. Two popular variations of RNNs are long short-term memory (LSTM) [20] and gated recurrent unit (GRU) [21]. Ibrahim et al. [22] combined CNN and LSTM to build a deep temporal model for group activity recognition. They used a pre-trained AlexNet [23] to extract the complex image-based feature describing the spatial region around a person. Along the CNN, the first LSTM network is trained to get person-level actions and their temporal evaluation. Another LSTM network is subsequently trained to combine person-level data to comprehend activities throughout the entire video. He et al. [24] utilized the bi-directional LSTM, comprising two independent LSTMs, to learn both forward and backward temporal information for human activity recognition. Ballas et al. [25] proposed a GRU-based approach for human action recognition and video captioning tasks utilizing the visual percept extracted by VGG-16 [26]. Another two-stream network architecture is optical flow-based video classification [27,28]. One stream processes the spatial information from the RGB frames, while the other processes the temporal information from the optical flow. The outputs of these two streams are then fused to make the final classification. Building on the success of 2D CNNs, numerous studies have explored extending these architectures to 3D CNNs. Temporal 3D ConvNet (T3D) [29] and Inflated 3D CNN (I3D) [30] are such examples. This shift to 3D allows the model to capture both spatial and temporal information simultaneously, rather than relying on separate streams for each type of data. This unified approach holds promise for improved video analysis by inherently considering the interplay between what is happening in each frame (spatial) and how it unfolds over time (temporal). However, many 3D CNN-based frameworks have a high number of parameters and, therefore, need a large amount of training data. To address this, Qiu et al. [15] decomposed the 3D convolution into a 2D convolution for the spatial domain followed by a 1D convolution for the temporal domain, providing a more efficient and effective simulation of 3D convolutions. In our work, we also adopted (2 + 1)D convolution, instead of 3D convolution.

Video AI classification in healthcare is still evolving but holds significant potential for analyzing medical videos, such as endoscopies, surgeries, and patient monitoring footage, to detect abnormalities, track disease progression, and assist in surgical planning. Yin et al. [31] proposed a deep learning-based automatic Parkinson’s disease (PD) diagnosis method using videos, employing a 3D convolutional neural network (CNN) for PD severity classification and exploring transfer learning from non-medical datasets. Zhang et al. [32] proposed the SPAPNet system, which classifies Parkinson’s tremors using non-intrusive video recordings of human movements. It utilizes a novel attention module with a lightweight pyramidal channel-squeezing fusion architecture, achieving a balanced accuracy of 90.9% and an F1-score of 90.6% in distinguishing Parkinson’s tremors from non-tremors. Li et al. [33] proposed TrackletNet, which combines a lightweight CNN and LSTM for the binary classification of tracklet clips related to lung consolidation and pleural effusion in ultrasound videos. For pleural effusion with only video-level annotations, Shea et al. [34] used an LSTM-CNN for binary classification. For consolidation and B-lines, with frame-level annotations, they applied a two-step classifier: the first step assessed individual frame confidence, and the second step aggregated these scores for video-level classification. Thuwajit et al. [35] proposed EEGWaveNet, a multiscale convolutional neural network that uses trainable depth-wise convolutions to analyze and extract spatial-temporal features for detecting seizures in epileptic patients using EEG. Krishnaswamy et al. [36] proposed a two-stream neural network to classify lung ultrasound videos into normal, interstitial abnormalities, and confluent abnormalities achieving an F1-score of 0.86. Jin et al. [37] proposed MTRCNet-CL, a multi-task recurrent convolutional network for surgical tool presence detection and surgical phase recognition from surgical videos. The model uses shared feature encoders and LSTM for temporal dependencies.

There is limited literature available on AI-based classification from endoscopic videos. Moreover, some studies [38,39] did not consider the temporal relationships between frames and treated them individually, thus falling into the 2D spatial domain. Though Ozturk et al. [5] incorporated LSTM with CNN, they trained and evaluated the model on image data, not video data. An earlier attempt was made in 2007 when Lee et al. [40] proposed an algorithm to discriminate digestive organs and the colon in wireless capsule endoscopy (WCE) videos using an energy function in the frequency domain to characterize contractions and a high-frequency content function to segment videos into events. Billah et al. [41] proposed an automatic system for polyp detection, utilizing color wavelet and CNN features to train a linear SVM. Owais et al. [11] combined ResNet and LSTM to classify multiple GI diseases from endoscopy videos. Yu et al. [13] proposed GL-Net, which combines a graph convolutional network (GCN) with long short-term memory (LSTM) networks for the diagnosis of upper gastrointestinal disorders from esophagogastroduodenoscopy (EGD) videos.

The purpose of this study was to develop a novel spatiotemporal deep learning framework tailored for medical video analysis, with potential applications in prognosis, diagnosis, and treatment monitoring. Gastrointestinal endoscopic video classification is employed as a feasibility use case to demonstrate the framework’s effectiveness and broader relevance across various video-based clinical settings. In this paper, we present an attention-fused 3D CNN to classify upper and lower GI from endoscopic videos collected from the hyperKvasir dataset [3]. We implement a 3D parallel spatial and channel squeeze-and-excitation (P-scSE3D) and propose a new block called residual with parallel attention (RPA). To reduce computation, we use a (2 + 1)D convolution [14,15], which applies 2D convolution for the spatial domain followed by a 1D convolution for the temporal domain.

## 2. Methods

**Figure 1:**
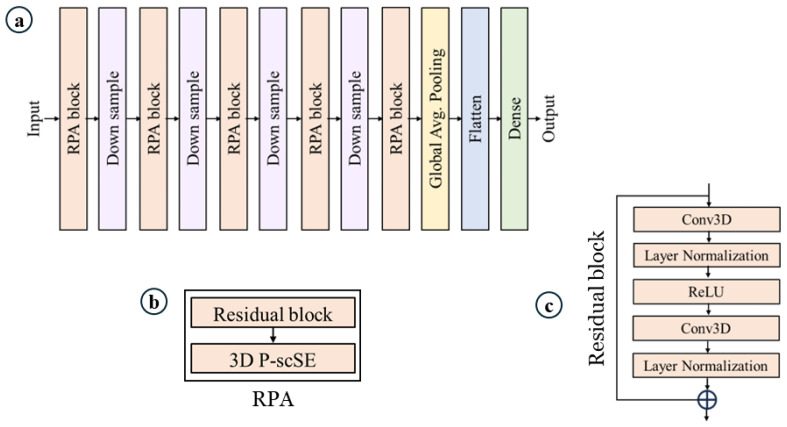
Proposed model. (**a**) Model architecture, (**b**) residual with parallel attention (RPA) block, which is the core of the model, and (**c**) residual block.

**Overview.** Figure 1 shows our proposed model. It is built on top of [42,43]. Our proposed model consists of a series of RPA blocks and downsample blocks. RPA stands for residual with parallel attention. Each RPA block consists of a residual block followed by a 3D parallel spatial and channel squeeze-and-excitation (P-scSE3D). In each downsample stage, the dimension is reduced by half. There are five RPA blocks and four downsample blocks. RPA blocks and downsample blocks create hierarchical feature maps. The classification head consists of a global average pooling layer, a flattening layer, and a dense layer. Global average pooling reduces the spatial dimensions of the data into a single value per feature channel. The flattening layer flattens the data into a one-dimensional vector. The dense layer is a fully-connected layer that performs linear regression on the data.

**Residual block.** As mentioned above, each RPA block consists of a residual block and a P-scSE3D. The residual block allows the network to learn from both the original input and the processed output, addressing the vanishing gradient problem that can occur in deep neural networks [44,45]. As depicted in Figure 1c, the input initially undergoes a convolution layer. However, instead of 3D convolution, we utilize (2 + 1)D convolution, which processes the spatial and temporal dimensions separately. This approach offers an advantage by reducing the number of parameters through the factorization of convolutions into spatial and temporal dimensions [15,43]. Subsequently, layer normalization is applied to normalize the outputs of the previous layer, which can improve training stability [44]. This activation function introduces non-linearity into the network by setting all negative outputs to zero and keeping the positive outputs unchanged. A ReLU [46] activation function is then employed to further introduce non-linearity into the network. It essentially sets all negative outputs to zero and keeps the positive outputs unchanged. We then reapply the (2 + 1)D convolution and layer normalization. Finally, the original input is added to the output of the second convolutional branch. This exemplifies the core concept of the residual block—it allows the network to learn the difference between the input and the output from the convolutional layers, potentially enabling it to capture more complex features. By incorporating residual blocks, the network can theoretically learn from both the original input and the refined features extracted through the convolutional layers, potentially improving the model’s performance.

**Figure 2:**
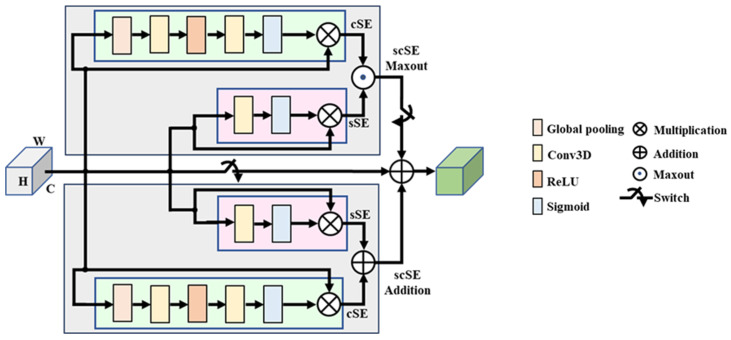
Parallel spatial and channel squeeze-and-excitation (P-scSE) module [47,50].

**P-scSE3D.** Parallel spatial and channel squeeze-and-excitation [47] was initially introduced for foot ulcer segmentation. The P-scSE module is an improvement upon the squeeze-and-excitation module [48,49], which aims to enhance a network’s representational capabilities by emphasizing important features while reducing the emphasis on less relevant ones. The original P-scSE3D was designed for 2-dimensional space. In this work, we extend it to 3-dimensional space. As shown in Figure 2, it consists of three main blocks: cSE, sSE, and scSE; cSE stands for spatial squeeze and channel excitation. As the name implies, it first squeezes spatial dimensions by applying global pooling and then excites channels by performing dimension reduction specified by a reduction ratio, *r*, that determines the capacity and computational cost of the cSE block. The dimension is increased back and maps to between 0 and 1 using a sigmoid function. These weights are then used to recalibrate the original input by performing an element-wise multiplication between them. The sSE block stands for channel squeeze and spatial excitation. Channels are squeezed by applying a convolution layer. Then, the excitation matrix is generated by applying a sigmoid function. This matrix is used to recalibrate the input the way it did in the cSE block. The cSE and sSE blocks are then combined to create the spatial and channel squeeze-and-excitation (scSE) block. The combination can be made in different ways such as adding them element-wise, taking the maximum between them (max-out), multiplying them, or concatenating them. The P-scSE module tackles the challenge of choosing the proper combination operation in emphasizing informative features. While the multiplication of outputs from two squeeze-and-excitation (SE) blocks seems intuitive, it risks losing crucial data, especially in healthcare where subtle details are essential. This happens because multiplication essentially mutes pixels needing emphasis from both blocks. Concatenation, on the other hand, preserves all information but doubles the number of channels, making the model computationally expensive. The P-scSE offers a clever solution: it uses parallel branches. One branch leverages max-out to ensure important features from either SE block are captured, while the other branch uses addition that gives equal importance to both blocks. The outputs from these branches are then simply added, achieving the benefits of both max-out and addition without further adjustments, as each SE block has already refined the data. A switch (SW) is utilized to configure the shorted P-scSE by bypassing the max-out scSE when there are fewer feature maps available.

## 3. Design Procedure

**Figure 3:**
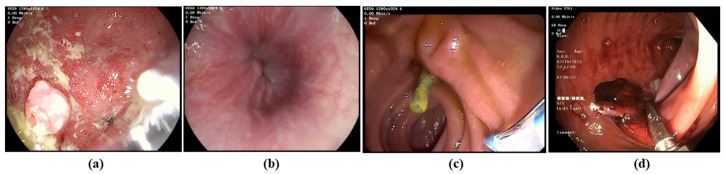
Representative examples of (**a**,**b**) upper GI image frame from endoscopic video, and (**c**,**d**) lower GI image frame from endoscopic video (Bottom).

**Dataset.** We utilized the HyperKvasir dataset [3] for our upper and lower gastrointestinal (GI) tract classification task. This dataset is rich in content, comprising 373 videos that display a wide array of findings and landmarks relevant to gastrointestinal examinations. In total, these videos amount to approximately 11.62 h of video footage, spanning a vast collection of 1,059,519 frames. Within this dataset, there is a notable distribution of 314 lower GI videos and 60 upper GI videos, each recorded at two different resolutions: 1280 × 1024 and 720 × 576. However, we preprocess all the videos to a fixed size of 224 × 224. Figure 3 demonstrates some samples of upper and lower GIs.

**Figure 4:**
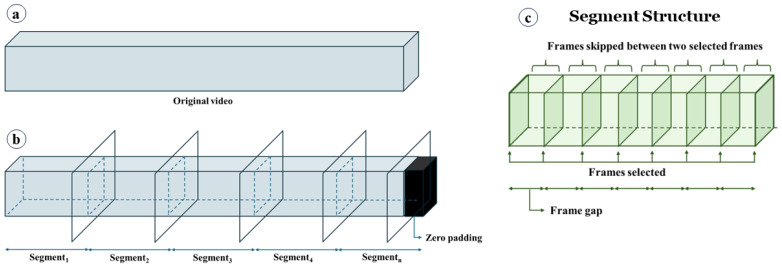
Video segment generation. (**a**) Original video, (**b**) video split into segments. Zero-padded frames are added to keep the segment size fixed, and (**c**) the structure of a segment. A frame gap is used to skip some frames.

**Generation of video segments.** The HyperKvasir dataset contains videos ranging in length from 1 s to over 12 min. Due to this varying length, it is not feasible to directly feed the original videos to the deep learning model. Therefore, we divide the original videos into segments (see Figure 4). As shown in Equation (1), each segment has a fixed size calculated by multiplying the frame gap (*G*) by the number of frames per segment (*N*). Instead of using consecutive frames, as illustrated in Figure 4c, we select frames at regular intervals defined by *G*. Hence; the frame gap is the stride used to select the next frame. Segments are generated on the fly during training to conserve memory. For example, if *G* = 15 and *N* = 10, the segment will consist of 10 frames, with each subsequent frame selected by skipping 15 frames from the previous frame (i.e., *Frame*_*i*+1_ − *Frame_i_* = 15). The rationale behind setting a frame interval is that consecutive frames do not vary significantly in terms of unique information, whereas selecting frames at regular intervals provides more diverse information while maintaining the same number of frames. During training, segments are randomly generated from the original videos, meaning that a segment can be any part of the video. In some cases, when segments are generated from the end of the videos, they may not contain enough frames. In such instances, zero-padded frames are inserted to maintain a constant segment size (see Figure 4b). Additionally, we found it unnecessary to consider all possible segments from a video to make predictions. To expedite the process, during inference, we consider a maximum of 10 segments for prediction. If a video has fewer than 10 segments, we consider the maximum number of segments possible. The segments are selected randomly. The impact of different frame gaps and the number of frames is discussed in later sections.

(1) $$Segment\;size\left(S\right)=Frame\;gap\;\left(G\right)\times No.\;of\;frames\;per\;segment\left(N\right),$$

**Training and inference.** Due to a significant imbalance in the number of upper and lower GI videos, we use all 60 upper GI videos and randomly sampled 60 lower GI videos in each experiment to ensure balanced training. Furthermore, to reduce sampling bias, we conduct 20 independent runs, each using a different random subset of the lower GI videos. For consistency across configurations, we fix the random seed such that the same 20 subsets are reused across all configurations. We do not need to randomly select videos from the upper GI category, as it contains exactly 60 videos. The final result is the average of these 20 runs. We use a split ratio of 70:15:15 for training, validation, and testing, respectively. Thus, there are 84 videos for training, 18 for validation, and 18 for testing in each experiment. We collect raw logits as the model’s output and use Keras’ *SparseCategoricalCrossentropy* loss function with from_logits = True. This setting ensures that Keras internally applies the softmax operation during loss computation for improved numerical stability. We use Adam optimizer [51] with an initial learning rate of 1 × 10^−4^. The batch size is set to 2, meaning we feed two segments to the model in each iteration. We train the model for 50 epochs in each experiment. All experiments are executed on an NVIDIA Tesla V100 GPU provided by Google Colab Pro+, with a capacity ranging from 16 GB to 32 GB, depending on availability. Codes are implemented in TensorFlow.

**Evaluation metrics.** For evaluation, we use the widely used accuracy, precision, recall, and F1-score. Here are the details pertaining to each definition:

(2) $$Accuracy=\frac{TP+TN}{TP+TN+FP+FN}$$

(3) $$Precision=\frac{TP}{TP+FP}$$

(4) $$Recall=\frac{TP}{TP+FN}$$

(5) $$F1-\mathrm{Score}=2\times \frac{Recall\times Precision}{Recall+Precision}$$

## 4. Results and Discussion

**Evaluation results.** We conducted experiments 20 times for each configuration using our proposed model to classify upper and lower gastrointestinal (GI) videos. Configurations were based on different numbers of frames (*N*) and frame gaps (*G*), designed to observe the impact of increasing *N* and decreasing *G*. For inference, we considered a maximum of 10 segments to make predictions. If a video was too small to have 10 segments, we considered all possible segments to extract from the video, which accelerated the testing process. Table 1 summarizes the model’s performance, evaluated using metrics such as accuracy, precision, recall, F1-score, and area under the curve (AUC), by averaging the results of 20 experiments. In addition, 95% confidence interval (CI) is also reported for the accuracy and F1-score. The evaluation metrics showed a roughly 2% change across all configurations, indicating that the model’s ability to identify relevant features was not significantly affected by the number of frames or frame gap within the tested range. However, there is a trade-off to consider: training time. As the number of frames increased and the frame gap decreased, training time significantly increased. This is likely because the model needed to process more data with more complex configurations. We observed that setting a small *G* with a large *N* did not provide significant benefits.

**Figure 5:**
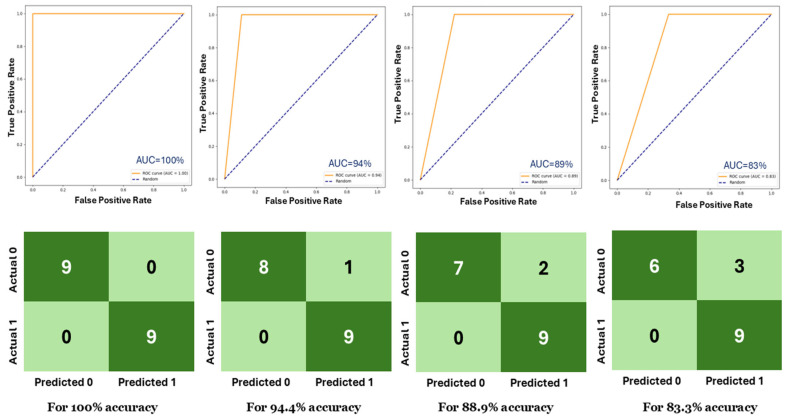
(**top**) ROC curves and (**bottom**) confusion matrices for different test accuracies.

For example, with *N* = 50 and *G* = 5 (resulting in a segment size of 250), the model achieved an F1-score of 93.5%. However, the same result could be achieved with a smaller segment size of 150 by using *N* = 10 and *G* = 15. This is due to the nature of the videos, where consecutive frames do not contain significant variation, and distant frames offer more distinct feature information. We also tabulated the maximum and minimum evaluation results found in 20 experiments. As shown in Table 2, all four configurations achieved 100% accuracy, F1-score, and AUC. Except for the (*N* = 100, *G* = 2) configuration, the other configurations achieved 83.3%, 82.4%, and 83.3% for accuracy, F1-score, and AUC, respectively. The (*N* = 100, *G* = 2) configuration showed the minimum performance among them. Table 3 tabulates the number of incorrect predictions for all 20 experiments. For instance, out of 20 experiments, there are 5 where the (*N* = 10, *G* = 15) configuration generated no incorrect predictions. In nine experiments, it made only one incorrect prediction. In three experiments, it made two incorrect predictions, and in three experiments, it made three incorrect predictions. It never made more than 3 incorrect predictions among all 20 experiments. Although the (*N* = 100, *G* = 2) configuration has seven experiments where it did not make any incorrect predictions, it made four incorrect predictions in four experiments. Considering the results of these three tables, we found the most optimal configuration is (*N* = 10, *G* = 15). This configuration will be used in the later discussion. Figure 5 displays the receiver operating characteristic (ROC) curve and confusion matrix for different test accuracies for (*N* = 10, *G* = 15), with the area under the curve (AUC) value ranging from 100% to 83%.

**Figure 6:**
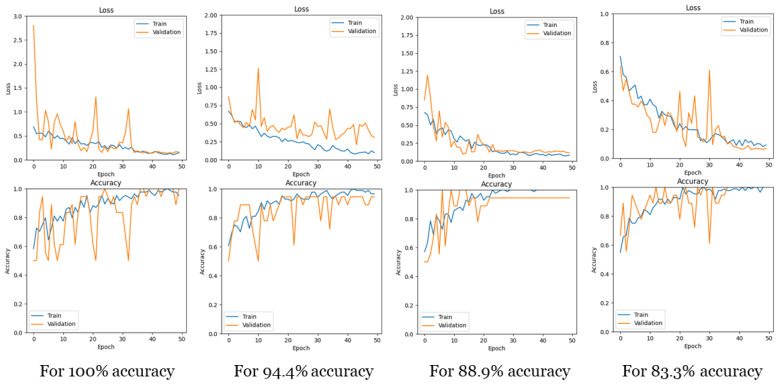
Loss and accuracy curves for training and validation for different test accuracies.

**Loss and accuracy curves.** Figure 6 illustrates the training and validation curves for loss and accuracy with (*N* = 10, *G* = 15), showcasing curves for different test accuracies. It is evident that for all test accuracies, the training proceeded without overfitting, as both the training and validation curves exhibited similar changes simultaneously.

**Explainable AI (XAI).** Explainable AI is a field in AI that focuses on making AI models more transparent. It helps us understand how AI systems reach their conclusions, particularly when those decisions are important. This is achieved by developing techniques that explain the reasoning behind AI outputs. As our model is a convolutional neural network (CNN) architecture, we used gradient-weighted class activation mapping (Guided Grad-CAM) [52] to visualize and understand the decisions made by the model. It helps in understanding which parts of data are important for the model’s prediction. The Grad-CAM technique generates a heatmap that highlights the regions of the input image that contributed the most to the final prediction. It does this by computing the gradient of the predicted class score with respect to the feature maps of the last convolutional layer.

**Figure 7:**
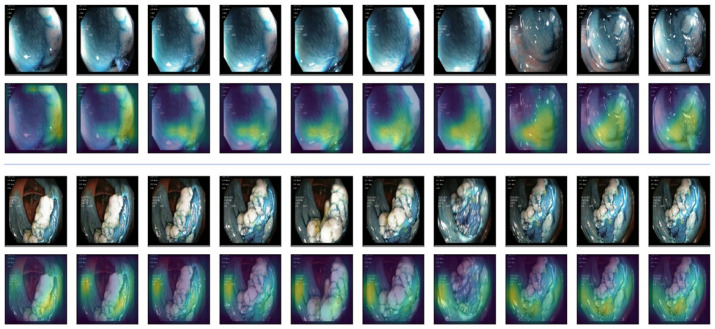
Explainable AI (XAI). Guided Grad-CAM is used as XAI. The 1st and 3rd rows indicate the original videos. The 2nd and 4th rows blend the heatmap on them. The yellow zone indicates the more focused zone used for classification.

These gradients are then used to weigh the importance of each feature map, and the heatmap is created by taking a weighted sum of the feature maps, followed by a ReLU activation. In Guided Grad-CAM, the gradients are passed backward through the network, but only positive gradients are allowed to propagate. This supposedly refines the Grad-CAM heatmap to focus on image features that directly contribute to the class activation. Figure 7 displays heatmaps for our GI data alongside the original frames for better comparison. In the samples, the yellow region indicates the more focused zone for making predictions. It is observed that the model successfully avoided the black border region and instead focused on the gastrointestinal regions.

**Effect of P-scSE3D.** We also investigated the effect of P-scSE3D. To assess its impact, we compared the results with and without P-scSE3D. In the ‘without P-scSE3D’ scenario, instead of RPA blocks, which contain both residual blocks and P-scSE3D, we used only residual blocks. Otherwise, both configurations share the same architecture, including identical depth and width. Table 4 summarizes the evaluation results for both scenarios with and without P-scSE3D for the (*N* = 10, *G* = 15) configuration. We observed a significant improvement after integrating P-scSE3D. For instance, the F1-score increased by 7% compared to the scenario without P-scSE3D. We extended the assessment by running both scenarios 50 times and checked the number of incorrect predictions made by each scenario. As shown in Table 5, the maximum number of incorrect predictions found for P-scSE3D is three, whereas it is seven for scenarios without P-scSE3D. Additionally, in 12 out of 50 runs, P-scSE3D predicted zero misclassifications.

To verify the statistical significance of the observed performance improvement, we conducted both a paired *t*-test and a Wilcoxon signed-rank test on the accuracy values obtained over 50 independent runs. The results (*t* = 2.64, *p* = 0.011; *W* = 184.5, *p* = 0.019) confirm that the improvement achieved by incorporating P-scSE3D is statistically significant (*p* < 0.05), supporting the effectiveness of the proposed module.

Our contributions can be summarized as follows:

*i*. **Novel Framework:** We developed a novel framework by introducing a 3D version of the parallel spatial and channel squeeze-and-excitation (P-scSE3D) module to a 3D CNN-based architecture tailored for classifying upper and lower GI endoscopic videos. This approach leverages spatiotemporal features to improve accuracy and efficiency.
*ii*. **Extensive experiments:** We conducted extensive experiments to demonstrate the potential of video-based studies in AI for GI endoscopy. The results show that the integration of the P-scSE3D module increased the F1-score by 7%.
*iii*. **Future Directions:** Our contributions lay the groundwork for future research for video AI, demonstrating its potential in GI endoscopy, including further optimization of 3D CNN architectures, exploration of additional clinical applications, and integration of explainable AI techniques for better interpretability. Additional wide-range video AI applications in retinal endoscopy, rhinoscopy, cardiac MRI and CT cine imaging applications, ultrasound videos in many disciplines, etc., will open new avenues for novel diagnostics leveraging this technology.
*iv*. **Ensuring Reproducibility:** Our model uses publicly available data that allows other researchers to explore this methodology, ensuring the reproducibility of our results and facilitating further research and exploration in this area.

## 5. Conclusions

This feasibility study developed a 3D convolutional neural network (CNN) for classifying upper and lower gastrointestinal (GI) endoscopic videos using the hyperKvasir dataset. Additionally, a 3D version of the parallel spatial and channel squeeze-and-excitation (P-scSE) module was implemented and integrated into the model to enhance performance. The model achieved an average accuracy of 0.933, precision of 0.932, recall of 0.944, F1-score of 0.935, and AUC of ROC curve of 0.933. Integration of the P-scSE3D module increased the F1-score by 7%. The study highlights the potential of video-based studies in AI for GI endoscopy and demonstrates the importance of explainable AI techniques like Grad-CAM for understanding model decisions. The results indicate that the (*N* = 10, *G* = 15) configuration is optimal for this task. This preliminary work creates opportunities to explore different prospective studies based on GI endoscopic videos. A comprehensive comparison with recent transformer-based video models, including Video Swin-T, TimeSformer-S, and MobileViT-v2-3D, is planned as part of future work. Future technical advancements will concentrate on rapidly implementing 3D CNN with innovative architecture to seamlessly integrate with real-time endoscopic workflows in GI clinical practice. Subsequent clinical applications will aim to identify various diseases that can benefit from the spatiotemporal features of GI videos, such as enteric neurologic disorders.

## Figures and Tables

**Table 1:** Average evaluation metrics for 20 runs for different configurations. Bold values indicate the best scores.

No. of Frames (*N*)	Frame Gap (*G*)	Accuracy	Precision	Recall	F1-Score	AUC	Time (min)
10	15	**0.933** (CI: 0.907, 0.959)	**0.932**	0.944	**0.935** (CI: 0.910, 0.960)	**0.933**	**4.067**
20	8	0.914 (CI: 0.889, 0.938)	0.906	0.933	0.916 (CI: 0.893, 0.940)	0.914	6.810
50	5	**0.933** (CI: 0.910, 0.961)	0.924	**0.950**	**0.935** (CI: 907, 0.962)	**0.933**	13.797
100	2	0.925 (CI: 0.891, 0.959)	0.925	0.939	0.928 (0.897, 0.959)	0.925	25.579

**Table 2:** Ranges of evaluation metrics found over 20 runs for different configurations.

No. of Frames (*N*)	Frame Gap (*G*)	Max Incorrect	Accuracy		F1-Score		AUC	
			Max	Min	Max	Min	Max	Min
10	15	3	1	0.833	1	0.824	1	0.833
20	8	3	1	0.833	1	0.824	1	0.833
50	5	3	1	0.833	1	0.824	1	0.833
100	2	4	1	0.778	1	0.818	1	0.778

**Table 3:** Frequency count for different numbers of incorrect predictions calculated over 20 runs.

No. of Frames	Frame Gap	No. of Incorrect Predictions				
		0	1	2	3	4
10	15	5	9	3	3	0
20	8	3	6	8	3	0
50	5	6	7	4	3	0
100	2	7	4	6	1	2

**Table 4:** Comparison between with and without P-scSE3D. Bold values indicate the best scores.

P-scSE3D	Accuracy	Precision	Recall	F1-Score	AUC
No	0.88	0.90	0.88	0.87	0.88
Yes	**0.93**	**0.93**	**0.94**	**0.94**	**0.93**

**Table 5:** Frequency count for different numbers of incorrect predictions calculated over 50 runs, considering both with and without P-scSE3D.

P-scSE3D	Number of Incorrect Predictions							
	0	1	2	3	4	5	6	7
No	3	17	13	9	5	1	1	1
Yes	12	14	16	8	0	0	0	0

## Data Availability

Publicly available data can be downloaded from https://datasets.simula.no/kvasir/ (accessed on 17 July 2025).
